# Publisher Correction: SAIGE-GENE+ improves the efficiency and accuracy of set-based rare variant association tests

**DOI:** 10.1038/s41588-022-01220-x

**Published:** 2022-10-18

**Authors:** Wei Zhou, Wenjian Bi, Zhangchen Zhao, Kushal K. Dey, Karthik A. Jagadeesh, Konrad J. Karczewski, Mark J. Daly, Benjamin M. Neale, Seunggeun Lee

**Affiliations:** 1 Analytic and Translational Genetics Unit, Massachusetts General Hospital, Boston, MA USA; 2 Program in Medical and Population Genetics, Broad Institute of Harvard and MIT, Cambridge, MA USA; 3 Stanley Center for Psychiatric Research, Broad Institute of Harvard and MIT, Cambridge, MA USA; 4 Department of Medical Genetics, School of Basic Medical Sciences, Peking University, Beijing, China; 5 Center for Statistical Genetics, University of Michigan School of Public Health, Ann Arbor, MI USA; 6 Department of Biostatistics, University of Michigan School of Public Health, Ann Arbor, MI USA; 7 Department of Epidemiology, Harvard T. H. Chan School of Public Health, Boston, MA USA; 8 Institute for Molecular Medicine Finland, Helsinki Institute of Life Sciences, University of Helsinki, Helsinki, Finland; 9 Graduate School of Data Science, Seoul National University, Seoul, Korea

**Keywords:** Genome-wide association studies, Software

Correction to: *Nature Genetics* 10.1038/s41588-022-01178-w, published online 22 September 2022.

In the version of this article initially published, a footnote designating Wei Zhou, Wenjian Bi and Zhangchen Zhao as equally contributing authors was missing and has now been amended to the HTML and PDF versions of the article.

