# Supplementary material for: SAIGE-GENE+ improves the efficiency and accuracy of set-based rare variant association tests
Source: Nat Genet. 2022 Sep 22;54(10):1466–9. doi: 10.1038/s41588-022-01178-w (PMC9534766; doi:10.1038/s41588-022-01178-w)
Supplement: Supplementary file 1 — Supplementary Note, Figs. 1–6 and References. [file 41588_2022_1178_MOESM1_ESM.pdf]

nature portfolio

https://doi.org/10.1038/s41588-022-01178-w

Supplementary information

SAIGE-GENE+ improves the efficiency and accuracy of set-based rare variant association tests

In the format provided by the authors and unedited

Supplementary Note

A. Simulation studies for type I error evaluation

The phenotypes were simulated based on real genotypes of randomly selected L= 30,000 LD-pruned ($r^2 <$ 0.2) markers from the odd chromosomes with MAF $\geq$ 1% from the following logistic mixed model $logit(\pi_{i0}) = \alpha_0 + X_{i1} + X_{i2} + \sum_{j=1}^{L} \hat{G}_{ij}\beta$, where $\hat{G}_{ij}$ is the standardized genotype value for the jth marker of ith individual. β is the genetic effect size following $N(0, \tau/L)$, where $\tau = 1$, which is the variance component parameter. Two covariates, X_{i1} and X_{i2}, were simulated from Bernoulli(0.5) and N(0,1). π_{i0} is the probability for the ith individual being a case given covariates X_{i1} and X_{i2}, and random effects. The intercept α_0 was determined by given prevalence (i.e. case-control ratios). We repeated the simulation for 20 times for different disease prevalence: 0.3%, 1%, and 10%, respectively. For each phenotype set, a null logistic mixed model was fitted in Step 1 with covariates including the first 4 genetic principal components, which were estimated for all white British ancestry participants in the UK Biobank, X_1 and X_2.

B. Implementation of SAIGE-GENE+ to improve the computational efficiency

In order to further improve the computational efficiency, we utilized several approaches in the implementation of SAIGE-GENE+. 1. In Step 1 for fitting the null model, covariates are treated as offset which decreases the computation time (**Supplementary Table 4**). 2. When incorporating multiple MAF cutoffs and functional annotations, genotypes or dosages for all markers for each testing gene or region are read in at once and subsets are extracted for different tests. This largely reduces the redundancy for reading in genotype or dosages. 3. Score tests and SPA tests have been re-implemented in Rcpp, which reduces the overhead of switching between C++ and R. 4. For the tested set with q markers, as illustrated in the SAIGE-GENE paper, the q x q matrix $R^{1/2}G'P_sGR^{1/2}$ is used to approximate the G'PG, whose eigenvalues are used to obtain the statistics of the SKAT test. To reduce memory usage, we divide the q markers to several blocks and store the submatrices that are required to approximate G'PG in the hard disk, and then compute the corresponding submatrix of G'PG using each pair of the blocks. This approach to saving memory usage has been previously used in other programs, such as KING[4] for estimating sample relatedness.

C. Additional evaluation for set-based tests using the sparse GRM in the UK Biobank

A recently developed generalized linear mixed model method, fastGWA-GLMM[1], proposed using the sparse GRM to fit the null generalized linear mixed model for single-variants association tests in UKBB. In SAIGE-GENE+, we implemented this option to allow users to fit the null generalized mixed model using a sparse GRM. We applied this option to the UKBB WES data and compared the exome-wide association results to the results using the full GRM. Three different coefficients of relationship cutoffs 0.05, 0.0884 (up to 3rd degree of relationship) and 0.176 (up to 2nd degree of relationship) were applied to the sparse GRM (**Extended Data Fig. 9**). We observed that association p-values using a sparse GRM with different coefficients of relationship cutoffs are highly correlated with those using the full GRM for different binary traits (**Extended Data Fig. 9**) and quantitative phenotypes (**Supplementary Figure 2**). Step 1 to fit the null model required a much smaller computation time when using a sparse GRM (**Extended Data Fig. 8**). For example, with 150,000 samples, Step 1 required < 1 CPU min with sparse GRM (up to 3rd degree of relationship), while it took 11 CPU hours with full GRM estimated on-the-fly with 93511 genetic markers

(**Supplementary Table 2**). Since the sample relatedness in the UKBB is modest, the computational performance gain of using the sparse GRM can be modest in data sets with widespread sample relatedness.

D. Comparison with REGENIE2

We observed that Burden test p-values by SAIGE-GENE+ were highly concordant with p-values by REGENIE2 (Pearson's correlation R^2 = 0.99 for $-\log_{10}$(p-value)) (**Supplementary Figure 4**). We also compared the empirical computation cost of SAIGE-GENE+ and REGENIE2[2]. In Step 1 for fitting null models, SAIGE-GENE+ with a full GRM was more efficient than REGENIE2 (**Extended Data Fig. 8A and Supplementary Table 2**). Out of the five runs with 150,000 white British ancestry individuals that were randomly sub-sampled from the UK Biobank WES data for glaucoma (1,741 cases and 162,408 controls), the median computation time for Step 1 was 11 CPU hours in SAIGE-GENE+ and 36.5 CPU hours in REGENIE2 and the median memory usage was 5.4 Gb in SAIGE-GENE+ and 7.3 Gb in REGENIE2**.** Moreover, when a sparse GRM instead of a full GRM was used in Step 1, the time cost and memory usage in SAIGE-GENE+ dramatically dropped (< 1 min and 0.61Gb)**.** In Step 2, similar computation cost was observed in the two methods for Burden tests **(Extended Data Fig. 8B** and **Supplementary Table 3):** 8.8 CPU hours and 0.93Gb by REGENIE2 and 9.1 CPU hours and 0.97Gb by SAIGE-GENE+, which additionally output the p-values by the Cauchy combination or minimum p-value. SAIGE-GENE+ conducts SKAT-O test, while REGENIE2 conducts Burden tests only and does not allow for incorporating marker level weights. Although SKAT-O tests in SAIGE-GENE+ required nearly 6-7x more computation time (60 CPU hours) and 2x more memory (2.0 Gb) (**Extended Data Fig. 8B** and **Supplementary Table 3**), SKAT-O tests were more powerful than Burden tests with more significant p-values (**Extended Data Fig. 2**) and higher median Chi-square statistics (**Supplementary Table 8**) in all different simulation scenarios (**Supplementary Table 7**).

E. SAIGE-GENE+ and SAIGE comparison in *BRCA2* and *GCK*

We applied both methods to 37 self-reported binary phenotypes in the UKBB WES data using three different maximum MAF cutoffs: 1%, 0.1%, and 0.01% to 18,372 genes including all missense and LoF variants. *BRCA2* for breast cancer with MAF $\leq$ 0.1% had a p-value 7.62×10^{-8} in SAIGE-GENE+ and 1.65×10^{-3} in SAIGE-GENE. Similarly, we observed the gene *GCK* for diabetes with maximum MAF 0.1% had a more significant p-value (1.22×10^{-13}) in SAIGE-GENE+ than in SAIGE-GENE (p-value = 4.06×10^{-6}).

The associations between *BRCA2* and breast cancer are highly enriched in the ultra-rare LoF variants that tend to have the same effect directions, as was observed that the collapsed variant from 142 ultra-rare LoF variants had a p-value = 4.9×10^{-22} (https://ukb-200kexome.leelabsg.org/assoc/BRCA2/20001_1002). It is known that the Burden test is more powerful than the SKAT[3] test when most genetic variants in the test set are causal (having non-zero effects) with the same effect direction, whereas the SKAT test is more powerful when a small proportion of genetic variants are causal with inconsistent effect directions. Without collapsing in SAIGE-GENE, the Burden test p-value (0.000738) was more significant than the SKAT test p-value (0.114). But because only a small proportion of variants are causal, Burden test could still suffer from a low association power. With collapsing in SAIGE-GENE+, the SKAT test (p-value=2.67×10^{-8}) had a more significant p-value than the Burden test (p-value = 0.00452). This could be because the association signal is largely contributed by the collapsed ultra-rare variants and the SKAT test is more robust to the large proportion of non-causal variants in the test sets. Similarly, it was observed that the

association between the gene *GCK* and diabetes was driven by the collapsed ultra-rare variants https://ukb-200kexome.leelabsg.org/assoc/GCK/2443.

F. Simulation studies for power evaluation

Phenotypes were simulated based on the real genotypes G of randomly selected L= 30,000 LD-pruned (r^2 < 0.2) markers from the odd chromosomes with MAF ≥ 1% from the following logistic mixed model logistic mixed model $logit(\pi_{i0}) = \alpha_0 + X_{i1} + X_{i2} + \sum_{j=1}^{L} \hat{G}_{ij}\beta + \sum g_{iqk}\beta_{ci}$, where π_{i0} is the probability for the ith individual being a case given covariates and random effects, $\hat{G}_{ij}$ is the standardized genotype value for the jth marker of ith individual, and β is the genetic effect size following $N(0, \tau/L)$, where $\tau = 1$, which is the variance component parameter. Two covariates, X_{i1} and X_{i2}, were simulated from Bernoulli(0.5) and N(0,1). The intercept α_0 was determined by the prevalence 10%. We note that with this prevalence both SAIGE-GENE and SAIGE-GENE+ have well controlled type I error rates for Burden, SKAT, and SKAT-O tests.In addition to the 30,000 variants to simulate random effects, we select 10 genes as causal genes to simulate phenotypes and evaluate power. g_{iqk} is the genotype value for the qth variant in the kth gene of ith individual, β_{Ci} is a vector of the causal genetic effect sizes. Since functionally severe variants are likely to be causal variants with large effect sizes, we considered different proportions of causal variants and effect sizes by the functional annotations. Two different settings of proportions of causal variants across multiple functional annotations were used for variants that are not ultra-rare (MAC > 10): 1. 20% of LoF, 10% of missense, and 2% of synonymous and 2. 30% of LoF, 10% of missense, and 2% of synonymous. As functionally deleterious variants are more likely to be rarer, we assumed that the proportions of causal variants out of the ultra-rare variants (MAC ≤ 10) were 3 times higher than the proportions of causal ones out of the less rare variants (MAC > 10) across different functional annotations (**Supplementary Table 7**). Two settings of the absolute effect sizes for causal missense and synonymous variants were used, $|0.25\log_{10}(MAF)|$ and $|0.15\log_{10}(MAF)|$, respectively and the absolute effect sizes for causal LoF variants were set to be twice greater: $|0.5\log_{10}(MAF)|$ and $|0.3\log_{10}(MAF)|$. Two different settings of effect directions were used among causal variants, 1. all causal variants had the same effect direction 2. 100% of LoF, 80% of missense, and 50% of synonymous variants increased disease risk while the rest of the causal variants decreased disease risks. In the second setting (different association direction), since LoF variants are not likely to have different effect directions, we still assumed that the effect directions of all LoF causal variants were the same. In total, three scenarios with different settings of proportions of causal variants across multiple functional annotations and different settings of absolute effect sizes for causal variants were used (**Supplementary Table 7**). For each simulation scenario, we repeated the simulation for 100 times, resulting in 1,000 p-values (10 genes with each having 100 p-values.

G. Exome-wide analyses of 30 quantitative and 141 binary traits in the UK Biobank

We applied SAIGE-GENE+ to analyze 18,372 genes in the UKBB WES data with 160K white British ancestry individuals for 30 quantitative and 141 binary traits. Since the expected number of p-values < 2.5×10^{-6} under no association across all the phenotypes is 7.85, the false discovery rate is 0.014. Several known gene-phenotype associations have been identified (**Supplementary Table 9 and 10**). For binary phenotypes, examples include *BRCA1*, *BRCA2*, *CHEK2*, *PALB2*, and *SAMHD1* for breast cancer, *CNTNAP3B, CDKN2A* and *MITF* for melanoma, *IL33* for asthma, *GCK* for type 2 diabetes, and *LDLR* for ischemic heart disease. For quantitative traits, *MC4R* and *GIPR* were significant for body mass index, *CETP*, *LIPG*, and *LPL* for the HDL cholesterol, *LDLR* and *PCSK9* for the LDL cholesterol, and *MEPE* for heel bone mineral density. We also identified potentially novel gene-phenotype associations (**Supplementary Table 9 and 10**). For

binary traits, a pancreatic cancer susceptibility gene *NOC2L*[1] was significant for hypovolemia (p-value = 9.68×10^{-7}), *CYP21A2*, known for the 21-hydroxylase deficient congenital adrenal hyperplasia (CAH)[2], was associated with allergy/adverse effect of penicillin (p-value = 3.63×10^{-7}). Also for quantitative traits, *CHEK2*, known for breast cancer and sex hormone-binding globulin measurement[3], was associated with age at menopause (p-value = 4.51×10^{-17}), *IGLL5*, encoding an immunoglobulin lambda-like polypeptide, was associated with lymphocyte count (p-value = 1.50×10^{-12}), and *NANOG*, which mediates germline development[4] and is highly expressed in embryonic carcinoma[5], was associated with age at first live birth (p-value = 2.01×10^{-6}).

Supplementary Figure 1. -log10(p-values) of Burden, SKAT, and SKAT-O tests from SAIGE-GENE+ using a full GRM and a sparse GRM to fit the null models for binary traits in UKBB WES data.

MAF <= 0.01%

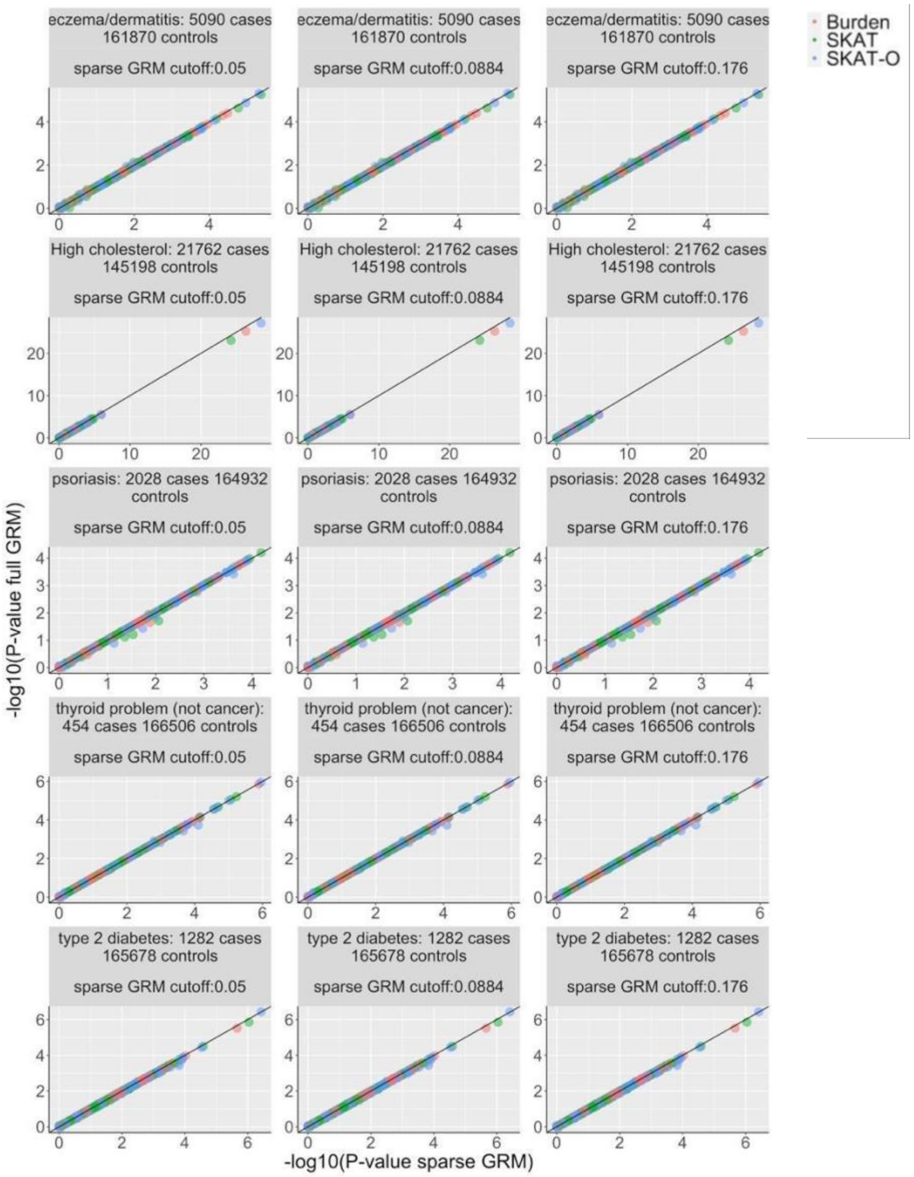

MAF <= 0.1%

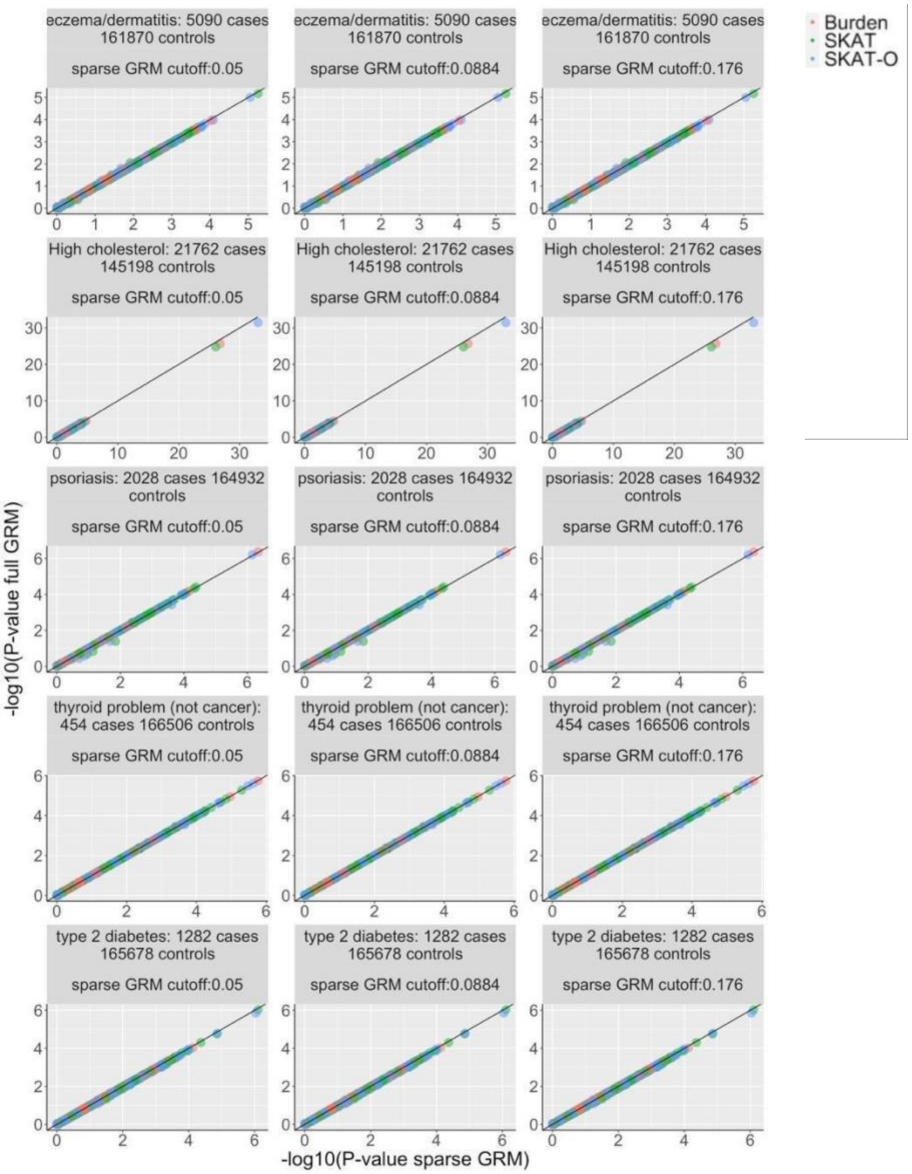
eczema/dermatitis: 5090 cases 161870 controls
sparse GRM cutoff:0.05
sparse GRM cutoff:0.0884
sparse GRM cutoff:0.176
High cholesterol: 21762 cases 145198 controls
psoriasis: 2028 cases 164932 controls
thyroid problem (not cancer): 454 cases 166506 controls
type 2 diabetes: 1282 cases 165678 controls
-log10(P-value full GRM)
-log10(P-value sparse GRM)
Burden
SKAT
SKAT-O

MAF <= 1%

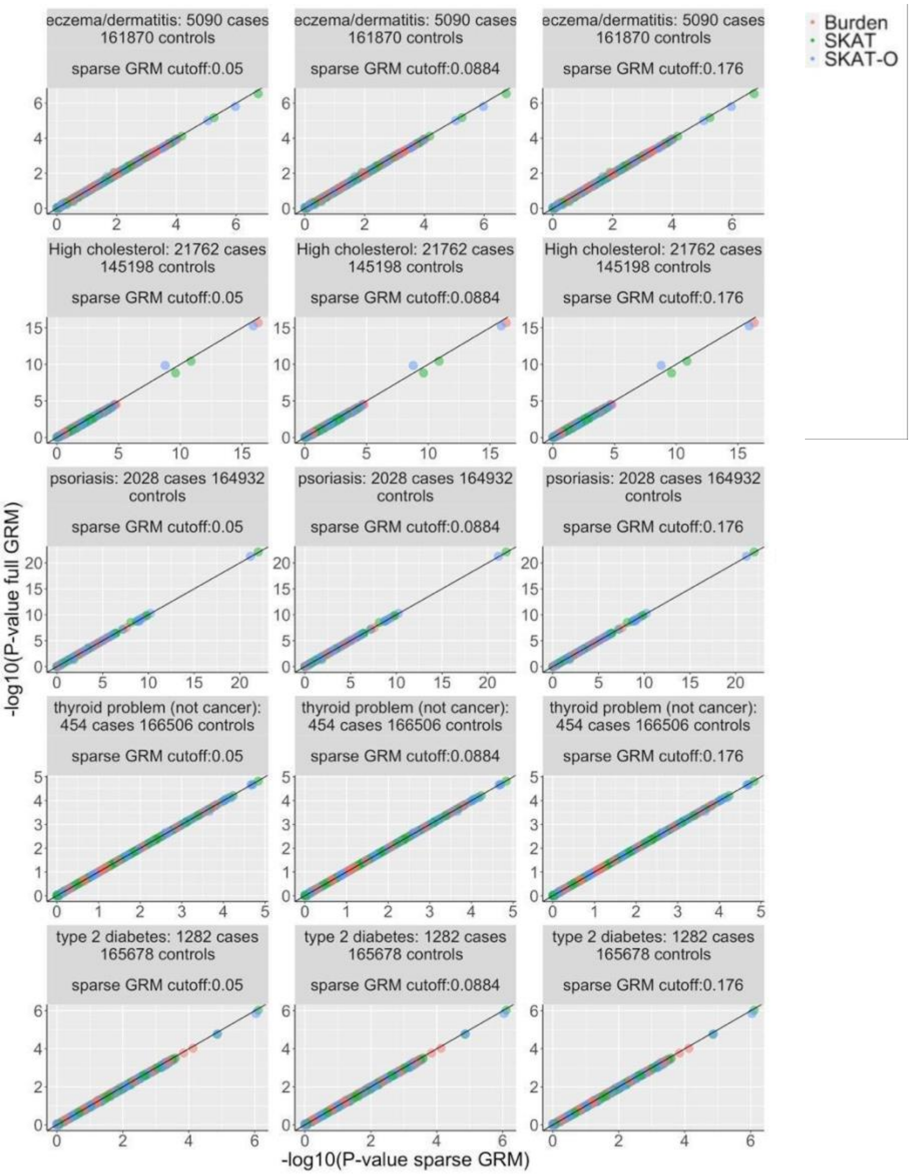
eczema/dermatitis: 5090 cases 161870 controls
sparse GRM cutoff:0.05
sparse GRM cutoff:0.0884
sparse GRM cutoff:0.176
High cholesterol: 21762 cases 145198 controls
psoriasis: 2028 cases 164932 controls
thyroid problem (not cancer): 454 cases 166506 controls
type 2 diabetes: 1282 cases 165678 controls
Burden
SKAT
SKAT-O
-log10(P-value full GRM)
-log10(P-value sparse GRM)

Supplementary Figure 2. -log10(p-values) of Burden, SKAT, and SKAT-O tests from SAIGE-GENE+ using a full GRM and a sparse GRM to fit the null models for quantitative traits in UKBB WES data

MAF <= 0.01%

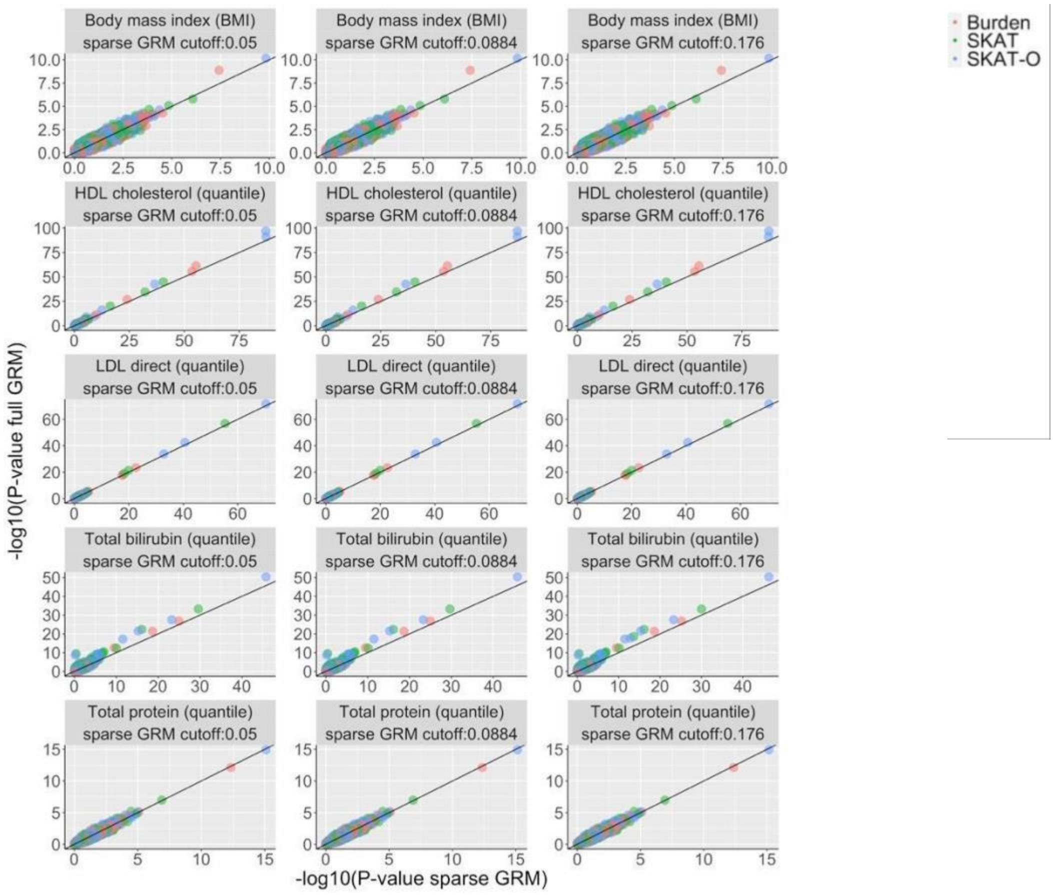

MAF <= 0.1%

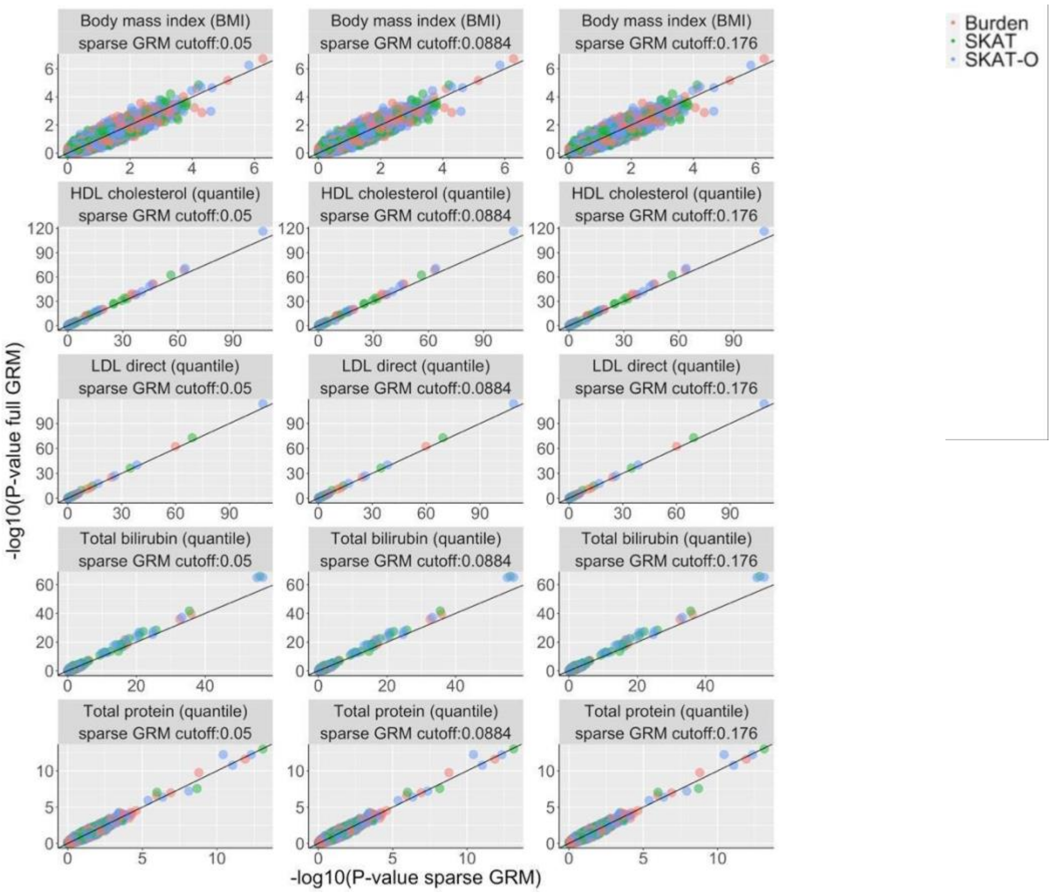
Body mass index (BMI)
sparse GRM cutoff:0.05
sparse GRM cutoff:0.0884
sparse GRM cutoff:0.176
HDL cholesterol (quantile)
LDL direct (quantile)
Total bilirubin (quantile)
Total protein (quantile)
-log10(P-value full GRM)
-log10(P-value sparse GRM)
Burden
SKAT
SKAT-O

MAF <= 1%

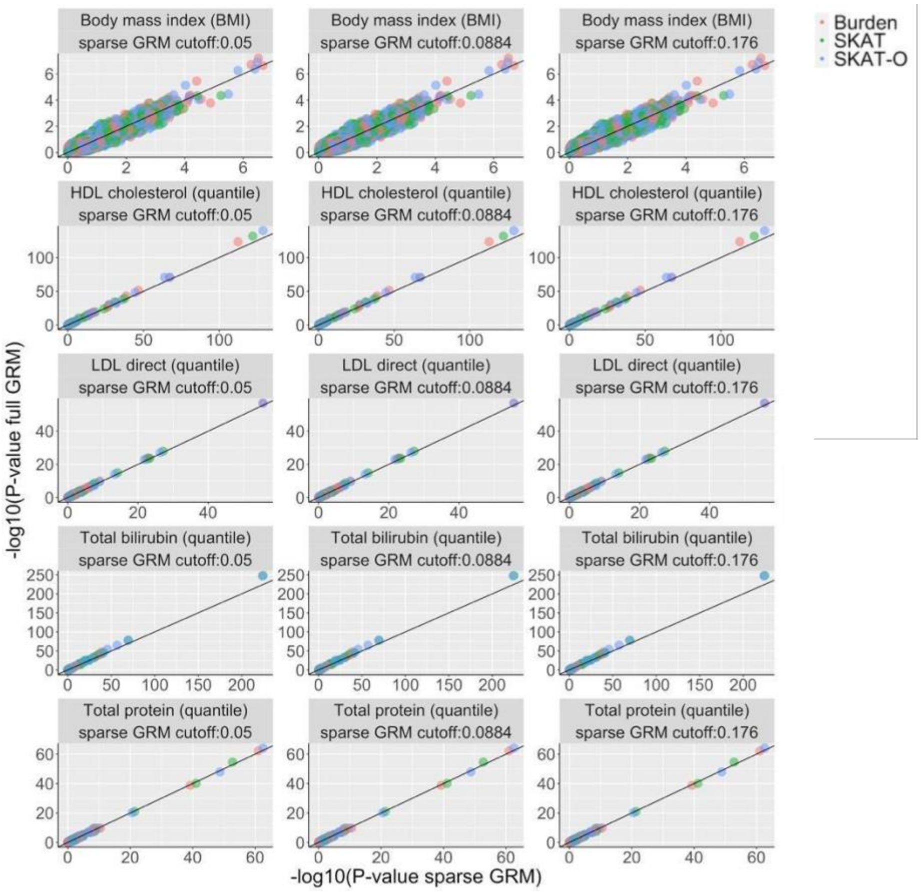
Body mass index (BMI)
sparse GRM cutoff:0.05
sparse GRM cutoff:0.0884
sparse GRM cutoff:0.176
HDL cholesterol (quantile)
LDL direct (quantile)
Total bilirubin (quantile)
Total protein (quantile)
-log10(P-value full GRM)
-log10(P-value sparse GRM)
Burden
SKAT
SKAT-O

Supplementary Figure 3. Scatter plots for association p-values of SKAT-O tests by SAIGE-GENE+ and SAIGE-GENE in simulation studies for power evaluation. Each plot is based on test results for 1,000 test sets (100 data sets, each of which includes 10 genes, see **Supplementary Table 6**). X-axis represents -log10 p-values without collapsing (SAIGE-GENE), and Y-axis represents -log10 p-values with collapsing (SAIGE-GENE+). The line in each plot represents the 45-degree line, so the dots above the line indicate more significant p-values from the collapsing. The details of different simulation settings are presented in **Supplementary Table 7**. A. All causal variants have the same effect direction. B. Causal variants have different effect directions (**Supplementary Table 7**).

A. Direction 1: All causal variants had negative effects

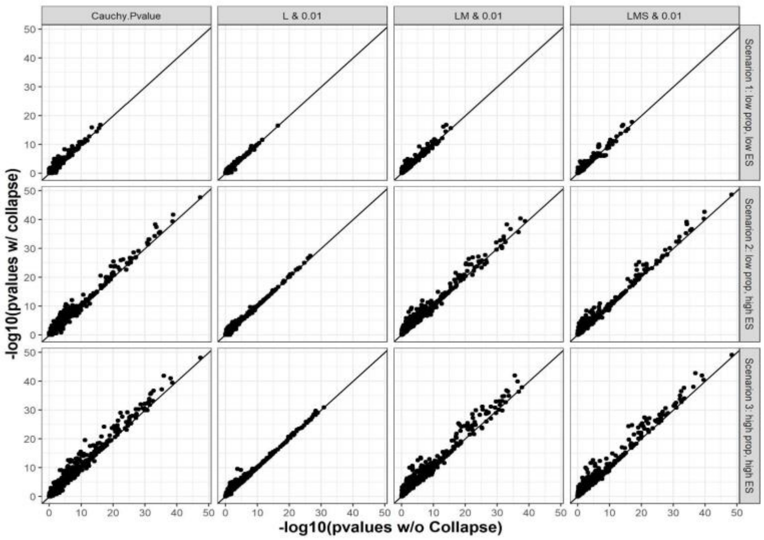

B. Direction 2: causal variants had different effect directions

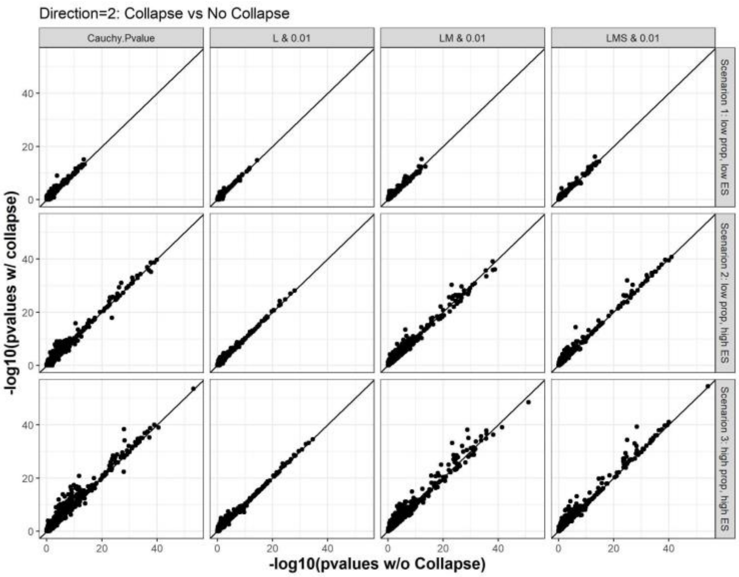

Supplementary Figure 4. Scatter plots for association p-values of Burden tests by SAIGE-GENE+ and REGENIE2. The default weights (Beta(MAF, 1, 25)) for genetic variants are used in SAIGE-GENE+ and the "sum" mask was used in REGENIE2. A. All causal variants have the same effect. B. Causal variants have different effect directions (**Supplementary Table 7**). Tests conducted in the analysis were two-sided.

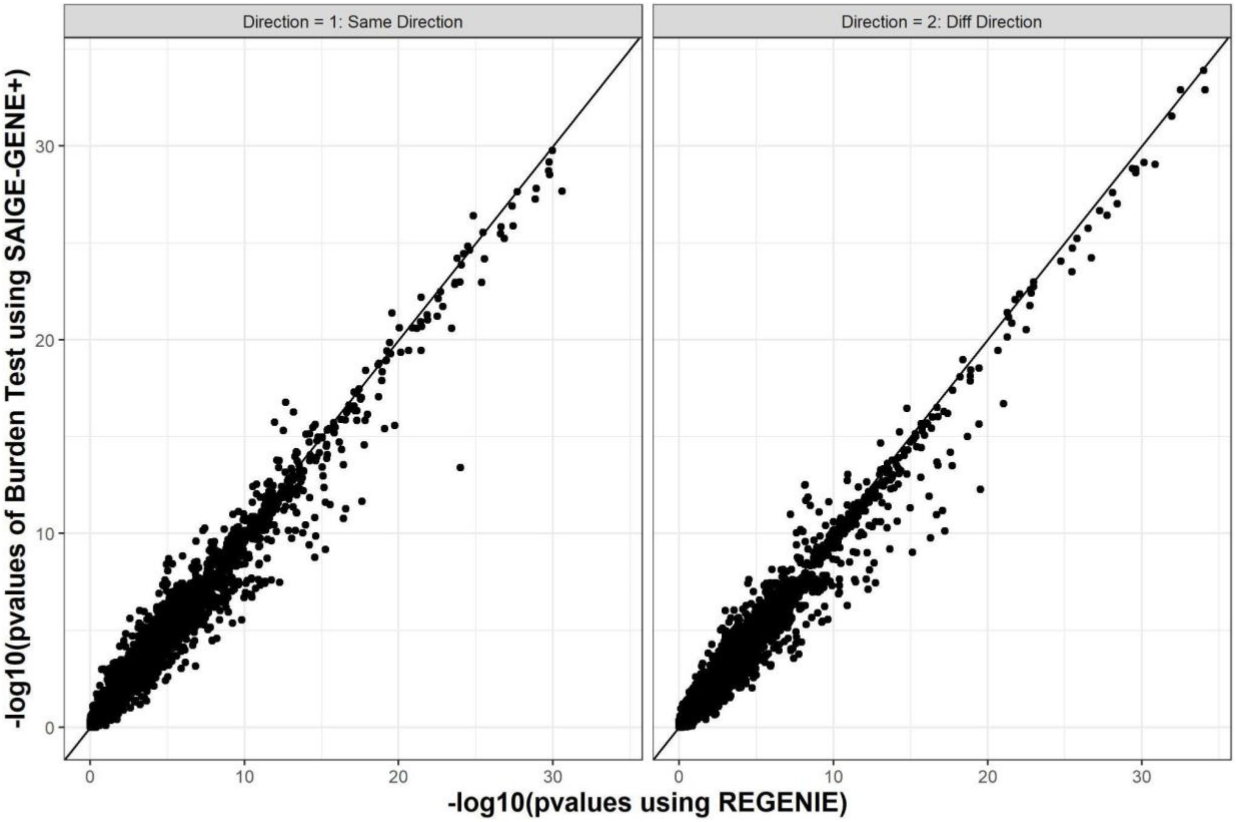

Supplementary Figure 5. Scatter plots for association p-values of SKAT-O tests in SAIGE-GENE+ using multiple MAF cutoffs and annotations and using a single cutoff and annotation. In the former tests, three different function annotation combinations (L only, L+S, L+S+M) and three maximum MAF cutoffs (0.01%, 0.1%, 1%) were used. In the latter tests, one function annotation and maximum MAF cutoff (L+S+M with MAF ≤ 1%) are used. The details of different simulation settings are presented in **Supplementary Table 7**.

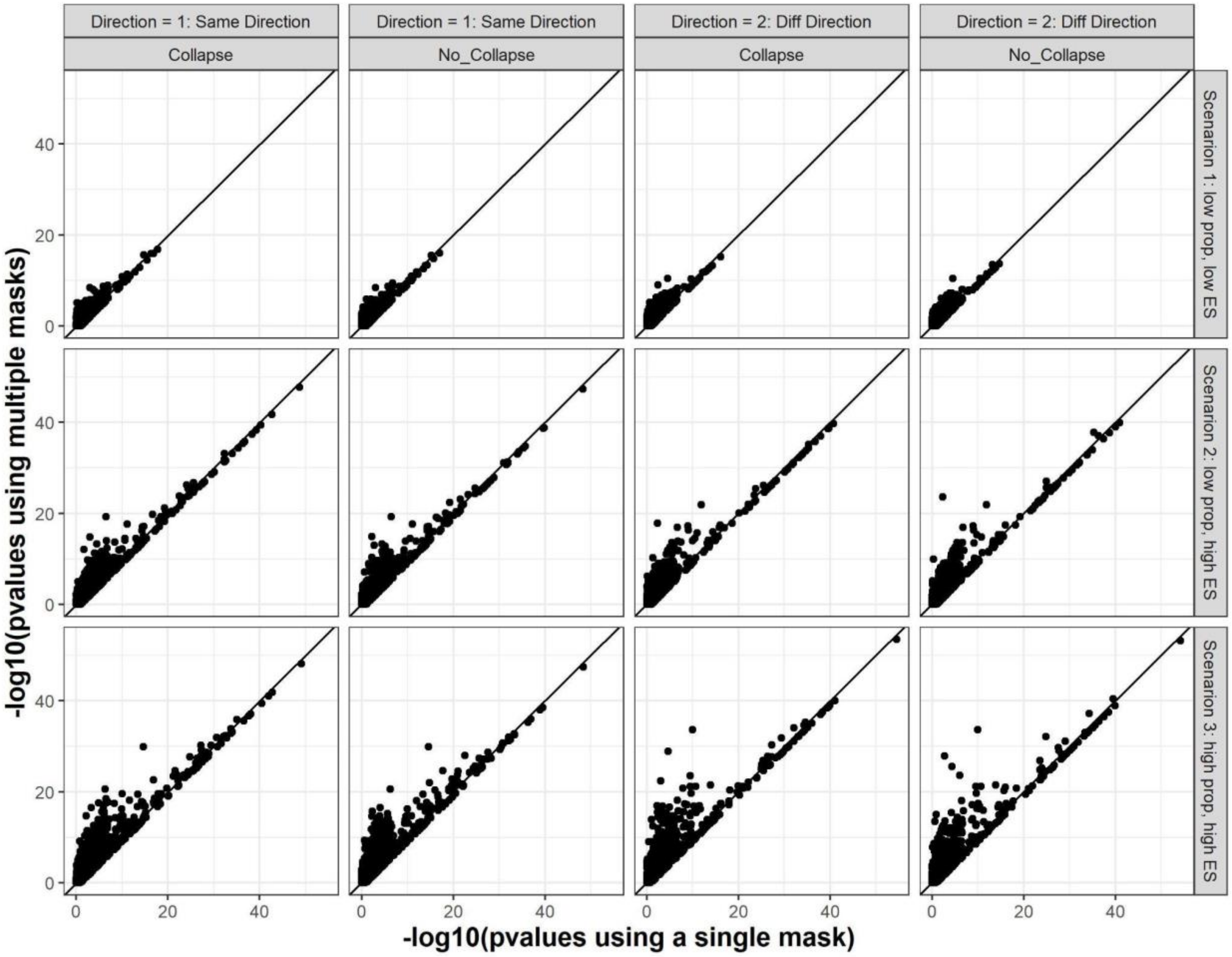

Supplementary Figure 6. Venn diagram of the number of associations identified (A) by different maximum MAF cutoffs (B) by different functional annotations

A.

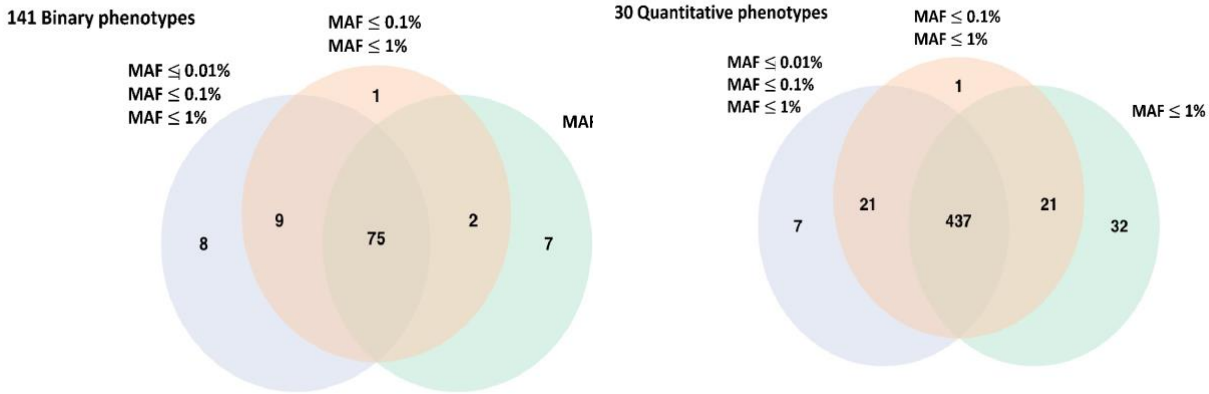

B.

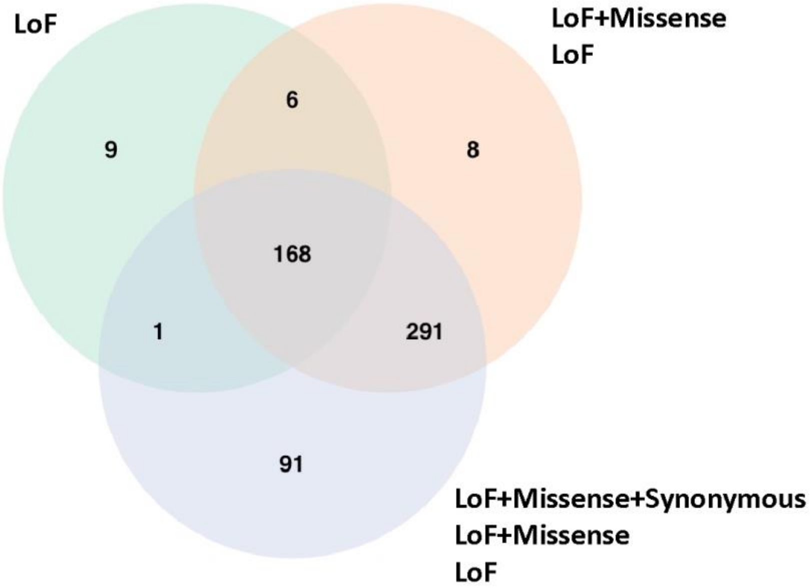

References

1. Klein, A. P. *et al.* Genome-wide meta-analysis identifies five new susceptibility loci for pancreatic cancer. *Nat. Commun.* **9**, 556 (2018).
2. Wedell, A., Ritzén, E. M., Haglund-Stengler, B. & Luthman, H. Steroid 21-hydroxylase deficiency: three additional mutated alleles and establishment of phenotype-genotype relationships of common mutations. *Proc. Natl. Acad. Sci. U. S. A.* **89**, 7232–7236 (1992).
3. Ruth, K. S. *et al.* Using human genetics to understand the disease impacts of testosterone in men and women. *Nat. Med.* **26**, 252–258 (2020).
4. Chambers, I. *et al.* Nanog safeguards pluripotency and mediates germline development. *Nature* **450**, 1230–1234 (2007).
5. Hart, A. H. *et al.* The pluripotency homeobox gene NANOG is expressed in human germ cell tumors. *Cancer* **104**, 2092–2098 (2005).
