# Supplementary material for: SAIGE-GENE+ improves the efficiency and accuracy of set-based rare variant association tests
Source: Nat Genet. 2022 Sep 22;54(10):1466–9. doi: 10.1038/s41588-022-01178-w (PMC9534766; doi:10.1038/s41588-022-01178-w)
Supplement: Supplementary file 2 — Reporting Summary [file 41588_2022_1178_MOESM2_ESM.pdf]

nature portfolio

Corresponding author(s): Seunggeun Lee

Last updated by author(s): Jul 13, 2022

Reporting Summary

Nature Portfolio wishes to improve the reproducibility of the work that we publish. This form provides structure for consistency and transparency in reporting. For further information on Nature Portfolio policies, see our Editorial Policies and the Editorial Policy Checklist.

Statistics

For all statistical analyses, confirm that the following items are present in the figure legend, table legend, main text, or Methods section.

n/a	Confirmed	
☐	☒	The exact sample size (*n*) for each experimental group/condition, given as a discrete number and unit of measurement
☐	☒	A statement on whether measurements were taken from distinct samples or whether the same sample was measured repeatedly
☐	☒	The statistical test(s) used AND whether they are one- or two-sided<br>*Only common tests should be described solely by name; describe more complex techniques in the Methods section.*
☐	☒	A description of all covariates tested
☐	☒	A description of any assumptions or corrections, such as tests of normality and adjustment for multiple comparisons
☐	☒	A full description of the statistical parameters including central tendency (e.g. means) or other basic estimates (e.g. regression coefficient) AND variation (e.g. standard deviation) or associated estimates of uncertainty (e.g. confidence intervals)
☐	☒	For null hypothesis testing, the test statistic (e.g. *F*, *t*, *r*) with confidence intervals, effect sizes, degrees of freedom and *P* value noted<br>*Give P values as exact values whenever suitable.*
☐	☒	For Bayesian analysis, information on the choice of priors and Markov chain Monte Carlo settings
☐	☒	For hierarchical and complex designs, identification of the appropriate level for tests and full reporting of outcomes
☐	☒	Estimates of effect sizes (e.g. Cohen's *d*, Pearson's *r*), indicating how they were calculated

Our web collection on statistics for biologists contains articles on many of the points above.

Software and code

Policy information about availability of computer code

Data collection	This research has been conducted using the UK Biobank Resource under application number 45227.
Data analysis	SAIGE-GENE(version0.44.6.1), https://github.com/weizhouUMICH/SAIGE/; SAIGE-GENE+ (version1.0.0), https://github.com/saigegit/SAIGE; STAAR (version0.9.5), https://github.com/xihaoli/STAAR; REGENIE(version2.2.4) https://github.com/rgcgithub/regenie.

For manuscripts utilizing custom algorithms or software that are central to the research but not yet described in published literature, software must be made available to editors and reviewers. We strongly encourage code deposition in a community repository (e.g. GitHub). See the Nature Portfolio guidelines for submitting code & software for further information.

Data

Policy information about availability of data

All manuscripts must include a data availability statement. This statement should provide the following information, where applicable:

- Accession codes, unique identifiers, or web links for publicly available datasets
- A description of any restrictions on data availability
- For clinical datasets or third party data, please ensure that the statement adheres to our policy

SAIGE-GENE+ is implemented as an open-source R package available at https://github.com/saigegit/SAIGE. The version used in the paper has been deposited at https://zenodo.org/badge/latestdoi/470322837. The summary statistics and QQ plots for 30 quantitative phenotypes and 141 binary phenotypes in UK Biobank by SAIGE-GENE+ are currently available for public download at https://storage.googleapis.com/leelabsg/saige-gene/reformat_all_withPhenoDetails.txt.

Field-specific reporting

Please select the one below that is the best fit for your research. If you are not sure, read the appropriate sections before making your selection.

☒ Life sciences ☐ Behavioural & social sciences ☐ Ecological, evolutionary & environmental sciences

For a reference copy of the document with all sections, see nature.com/documents/nr-reporting-summary-flat.pdf

Life sciences study design

All studies must disclose on these points even when the disclosure is negative.

Sample size	We analyzed publicly available UK Biobank whole exome sequencing data of white British samples (sample size=166,955). For the study design, please refer UK Biobank (http://www.ukbiobank.ac.uk/).
Data exclusions	To avoid confounding, analyses were done in white British samples in UK Biobank with non White British samples excluded.
Replication	For all exome-wide significant loci, we searched published GWAS studies to check whether they were known (replicated) or novel.
Randomization	We used publicly available UK Biobank data to illustrate the performance of the method. We randomly selected subsets of samples from the data set to evaluate method performance.
Blinding	We used coded public data, and hence were blinded.

Reporting for specific materials, systems and methods

We require information from authors about some types of materials, experimental systems and methods used in many studies. Here, indicate whether each material, system or method listed is relevant to your study. If you are not sure if a list item applies to your research, read the appropriate section before selecting a response.

Materials & experimental systems

n/a	Involved in the study	
☒	☐	Antibodies
☒	☐	Eukaryotic cell lines
☒	☐	Palaeontology and archaeology
☒	☐	Animals and other organisms
☐	☒	Human research participants
☒	☐	Clinical data
☒	☐	Dual use research of concern

Methods

n/a	Involved in the study	
☒	☐	ChIP-seq
☒	☐	Flow cytometry
☒	☐	MRI-based neuroimaging

Human research participants

Policy information about studies involving human research participants

Population characteristics	The UK Biobank study population (http://www.ukbiobank.ac.uk/) is residents of the UK aged 40-69 years at recruitment and living within a reasonable travelling distance of an assessment centre.
Recruitment	The UK Biobank participants were selected using the NHS register, and invited to volunteer for the study. Recruitment was carried out between 2007 and 2010. Full details of the recruitment process are available in reference (UK Biobank: Protocol for a large-scale prospective epidemiological resource, 2007).
Ethics oversight	National Research Ethics Service Committee (UK Biobank)

Note that full information on the approval of the study protocol must also be provided in the manuscript.
